# Prevalence and Risk for Bundle Branch Block, Atrioventricular Block and Pacemaker Implantation in Spondyloarthritis. A Systematic Review of the Literature

**DOI:** 10.3389/fmed.2022.851483

**Published:** 2022-03-25

**Authors:** Hye Sang Park, Ana Laiz, Petra Díaz del Campo, María A. Martín Martínez, M. Guerra-Rodriguez, Concepcion Alonso-Martin, Jesus Sanchez-Vega, Hector Corominas

**Affiliations:** ^1^Department of Rheumatology, Hospital Dos de Maig, Barcelona, Spain; ^2^Department of Rheumatology and Autoimmune Diseases, Hospital Universitari de la Santa Creu i Sant Pau, Barcelona, Spain; ^3^Department of Medicine, Universitat Autònoma de Barcelona (UAB), Barcelona, Spain; ^4^Unidad de Investigación, Spanish Society of Rheumatology, Madrid, Spain; ^5^Department of Cardiology, Hospital Universitari de la Santa Creu i Sant Pau, Barcelona, Spain; ^6^Department of Cardiology, Hospital Universitari Bellvitge, Hospitalet de Llobregat, Universitat de Barcelona (UB), Barcelona, Spain

**Keywords:** spondylarthritis, spondyloarthritis, cardiac conduction system disease, pacemaker, epidemiology, systematic review

## Abstract

**Objective:**

To evaluate the evidence regarding the prevalence and risk of bundle branch block (BBB), atrioventricular block (AVB) and pacemaker implantation (PMI) in patients with spondyloarthritis compared to a control group without spondyloarthritis.

**Methods:**

A systematic review of the literature was performed using Pubmed (Medline), EMBASE (Elsevier) and Cochrane Library (Wiley) databases until December 2021. The prevalence and risk for AVB, BBB and PMI were analyzed. Cohort, case control and cross-sectional studies in patients ≥18 years meeting the classification criteria for spondyloarthritis were included. The Odds ratio (OR), risk ratio (RR), or Hazard ratio (HR) and prevalence difference were considered as outcomes. Data was synthesized in a previously defined extraction form which included a risk of bias assessment using the Newcastle-Ottawa Scale.

**Results:**

In total, eight out of 374 studies were included. None of the studies provided results regarding the risk of low grade AVB and BBB in SpA patients. Only indirect results comparing prevalences from low to medium quality studies were found. According to population based registries, the sex and age adjusted HR of AVB was 2.3 (95% CI 1.6–3.3) in ankylosing spondylitis, 2.9 (95% CI 1.8–4.7) in undifferentiated spondyloarthritis and 1.5 (95% CI 1.1 a 1.9) in psoriatic arthritis. The absolute risk for AVB was 0.4% (moderate to high; 95% CI 0.34%-0.69%) for AS, 0.33% (moderate to high; 95% CI 0.21%-0.53%) for uSpA and 0.34% (moderate to high; 95% CI 0.26%-0.45%) for PsA.The RR for PMI in AS patients was 1.3 (95% CI 1.16–1.46) for groups aged between 65 and 69 years, 1.33 (95% CI 1.22–1.44) for 70–75 years, 1.24 (95% CI 1.55–1.33) for 75–79 years and 1.11 (95% CI 1.06–1.17) for groups older than 80 years. The absolute risk for PMI in AS patients was 0.7% (moderate to high risk; 95% CI 0.6–0.8%) for groups aged between 65–69, 1.44% (high risk; 95% CI 1.33–1.6%) for 70–75 years, 2.09% (high risk; 95% CI 2.0–2.2%) for 75–79 years and 4.15% (high risk; 95% CI 4.0–4.3%) for groups older than 80 years

**Conclusions:**

Very few cases of low grade AVB and BBB were observed in observational studies. No study evaluated association measures for low grade AVB and BBB but the differences of prevalence were similar in SpA and control groups even though studies lacked the power to detect statistical differences. According to population based registries there was an approximately two fold-increased risk of high grade AVB in SpA patients. RR for PMI was higher in younger age groups.

## Introduction

Over the last 100 years, cardiac vasculature, valves, myocardium, pericardium, and conduction system disorders were observed in inflammatory rheumatic disease. The cardiac diseases most commonly described were cardiovascular ischaemic events, valve diseases, ventricular dysfunction and conduction disorders (1). The common hypothesis is that systemic inflammation accelerates tissue degeneration which in turn causes cardiovascular diseases. Nevertheless, it is unknown whether the different inflammatory rheumatic diseases have tropism for a specific cardiac structure or tissue (1).

The aortic valve attachment site and peripheral entheses are known to share histological similarities (2). Two experimental studies observed that enthesis tissue resident T cells were also present in the aortic root and valve while being absent from the myocardium. Overexpression of interleukin-23 *in vivo* resulted in a dense infiltrate of T cells, macrophages and neutrophils in the attachment site of the aortic valve leading to the aortic wall, as well as in the enthesis (3, 4). According to those findings, inflammation of the valve attachment site may produce tissue degeneration near the atrioventricular node, which may lead to electrical conduction disturbancesdistrubances, that is to say atrioventricular block (AVB) and bundle branch block (BBB).

Among a sea of similar information, it is necessary to critically review the available evidence up-to-date and acknowledge the downsides to guide further investigation of each of the cardiac manifestations and inflammatory rheumatic diseases. We have previously carried out a systematic review of the literature of the prevalence and risk of heart valve and aortic involvement in spondyloarthritis (SpA) (5). Higher risk for valvular heart disease was observed in SpA patients and a with an approximately HR 1.97 adjusted by confounders and RR of 1.2. A a smallsmall increase was observed for aortic valve procedures RR 1.1–1.3 but not for mitral valve. Most studies were not adjusted by potential confounders.

The aim of this systematic review of the literature is to evaluate the prevalence and risk of bundle branch block (BBB), atrioventricular block (AVB) and pacemaker implantation (PM) in patients with spondyloarthritis compared to a control group without spondyloarthritis.

## Materials and Methods

A systematic review was conducted to identify all studies published up to December, 2021. This review was guided by the preferred reporting items for systematic review and meta-analysis (PRISMA) statements (Supplementary Table 1).

### Research Question

The research question was defined using PICO (Population, Intervention, Comparison, Outcome and Study design) components.

Are patients ≥ 18 years who have spondyloarthritis at an increased risk or higher prevalence for AVB, BBB and PMI compared with a control group that do not have spondyloarthritis?

### Inclusion Criteria

We included studies that met the following requirements:

- Study population: patients older than 18 years, diagnosed as Spondyloarthritis disease classified by ASAS, ESSG, Amor, modified New York, ARA, Moll & Wright or CASPAR criteria. Also patients diagnosed with spondyloarthritis following International Classification of Diseases (ICD) codes, or with a diagnosis confirmed by grade II bilateral sacroiliitis or grade III or superior if unilateral sacroiliitis was identified by X-Ray; and/or HLAB27 positivity; and fulfilling two or more clinical characteristics (peripheral arthritis, enthesitis, dactylitis, uveitis, inflammatory back pain, psoriasis, inflammatory bowel disease, history of urethritis or infective diarrhea) were included.
- Intervention: studies containing information about atrioventricular block, left and right bundle branch block measured by electrocardiogram (ECG) or holter.
- Outcome variables: studies containing information about association measures such as incidence ratio (IR), odds ratio (OR), risk ratio (RR) and Hazard Ratio (HR) and comparison of prevalences.
- Study design: Systematic review of the literature, meta-analysis, case control, cohort, cross sectional studies with more than 100 patients or population based registries.
- Language: English, French, Korean and Spanish.

### Exclusion Criteria

Studies carried out on pregnant women, animals or ethnic minorities. Abstracts, posters, narrative reviews, letters, editorials and any type of unpublished study.

### Search Strategy

A systematic search strategy was performed using the databases of Pubmed (Medline), EMBASE (Elsevier) and the Cochrane Library (Wiley) by a librarian (MG). The search strategy included MeSH terms and free text using different combinations (Supplementary Figure 1). Additional references were manually retrieved by reviewing the references of the included studies. Four studies were not available mainly because of antiquity (Supplementary Figure 2). They were previously consulted in two different national university library sources and the documentary collection of the Spanish Society of Rheumatology. An update of the systematic research was performed in December 2021 before the submission of this manuscript.

### Article Selection

A total of 373 citations were peer reviewed by two rheumatologists (HSP & AL). The reviewers independently performed two-stage screening (title/abstract and full-text screening), data extraction, and a risk of bias assessment. EndNote X8 software was used to manage the literature references. Those articles that were not considered relevant for further checking were excluded and the reason for exclusion were listed (Supplementary Figure 3). Senior methodologists (PDC & MAM) and a cardiac electrophysiologist (CAM & JSV) were consulted to retrieve clinically relevant data and for the interpretation of the results.

### Data Extraction and Data Analysis

For data extraction, a previously designed form in Word^®^ format was used. Data collected from each study were: country of study, design, sample size, participant selection, period of recruitment, follow-up period, method applied for conduction disorder diagnosis, magnitude of association and confounder factors. Prevalence odds ratio (POR), OR or comparison of proportions were calculated with the information available if necessary. Data was extracted by one reviewer (HSP) and supervised by another reviewer (AL). Disagreements between the reviewers were solved by discussion. When there was no consensus a third reviewer was consulted (PDC or CAM).

After gathering information, a narrative synthesis was preferred over a meta-analysis due to heterogeneity in population, design and/or the outcome measure of the included studies.

### Risk of Bias

The risk of bias was assessed by two reviewers (HSP & AL) using the Newcastle-Ottawa Scale (NOS) for assessing the quality of nonrandomized studies. The NOS contains eight items with an overall minimum score of one and a maximum of nine stars. There are three main quality dimensions: (1) selection of the study population; (2) comparability between the groups; and (3) outcome or exposure measures for cohort and case– control studies, respectively. The overall scores given were categorized into high (8–9 stars), medium (6–7 stars) and low quality (≤5 stars). We also categorized studies according to exposure assessment quality into high (three stars), medium (two stars) and low quality (one star). The complete checklist used is included in the Supplementary Material.

## Results

### Study Selection

**Figure 1:**
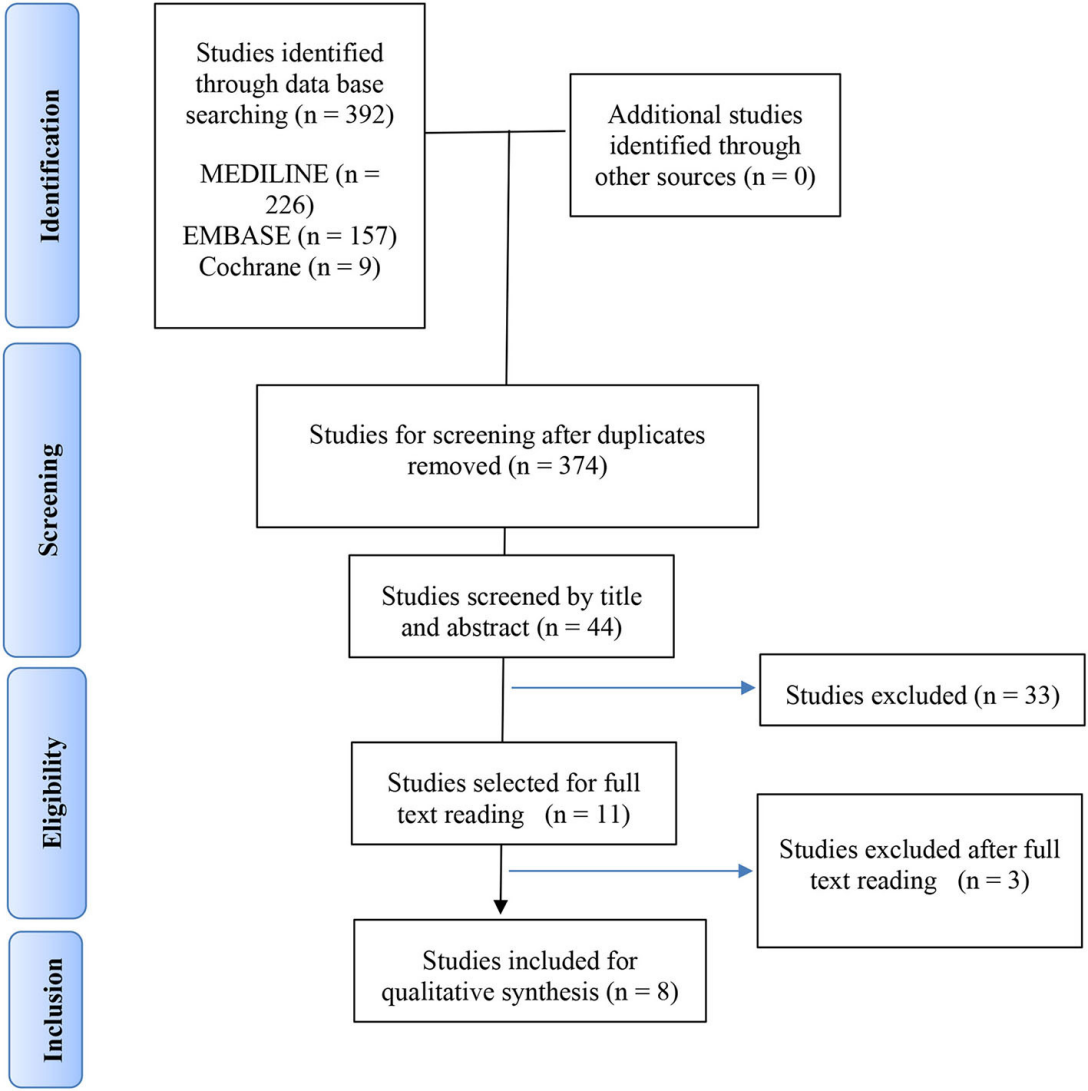
PRISMA flow chart.

We selected 62 studies of the original 374 studies for further reading based on title and abstract screening. After excluding 53 studies following full text reading, we included eight studies in the analysis, as presented in the PRISMA flow-chart (Figure 1). The reasons for excluding the remaining studies after reading the full text are provided in Supplementary Figure 3. No additional study was retrieved after reviewing the bibliography of the articles included for a full text reading. The final selection included two population based cohort studies, two case control studies and four cross-sectional studies.

### Characteristics of the Selected Studies

Detailed information regarding study design, location, sample size, follow up duration, exclusion and inclusion criteria, the methodology applied for the assessment of conduction disorder and outcome measures is summarized (Table 1).

**Table 1:** Characteristics of the selected studies.

**References**	**Study design**	**Population**	**Sample number**	**Follow up**	**Test**	**Outcomes**	**Adjustment**
Baniaamam et al. (6)	Cross sectional	AS and osteoarthritis between 50 and 75 years	267	-	ECG	Prevalence of AVB, BBB and PMI	Controls matched for age, sex and smoking status
Bengtsson et al. (7)	Cohort	AS, uSpA, PsA, GP from the Swedish national registry	294136	7 years (1^st^ Jan 2006–31st Dec 2012)	ICD-10	Prevalence, Incidence, HR, for AVB and PMI compared to GP	Age, sex
Dik et al. (8)	Cross sectional	AS	131	-	ECG	Prevalence of AVB and BBB Association of PR interval with AS disease related variables	Age, sex, disease duration
Feld et al. (9)	Case control	PsA compared to non psoriatic nor arthritic patients	184	Average 7.5 years (no further information)	ECG	Prevalence of AVB, BBB Correlation of PR interval with AS disease related variables	None
Fu et al. (10)	Cross sectional	AS between 18 and 50y without cardiac disease	122	-	ECG	Prevalence of AVB, BBB AS without kyphosis	None
Goulenok et al. (11)	Cross sectional	SpA, RA and control group without known CV disease	288	-	ECG	Prevalence AVB, BB	None
Ward (12)	Cohort	AS from Medicare database older than 65	42,327	14 years (1999–2013)	ICD-9	Prevalence, incidence, OR of PMI	Age, sex, race
Yildrir et al. (13)	Case control	AS	88		Holter and ECG	Prevalence of AVB	None

### Baseline Characteristics of the Participants

Mean age at enrollment was 33–54 years in five of the eight studies. Three studies were based on a specific age population: one study was based on patients between 50 and 75 years old (6), one study was based on patients older than 65 of age (12) and another study was based on patients between 18 and 50 of age (10). The proportion of women ranged from 8 to 72% depending on the type of spondyloarthritis. Mean disease duration was 3.9–22 years (6, 8, 9, 13). Hypertension was observed in 24.1–66% of patients (6–8, 11). Ischaemic heart disease was observed in 7.3–9% (6, 7, 9). Mean BASDAI was 3.1–5.9 (6, 8, 11). Uveitis was observed in 1.6–20.8% (7, 11, 13) depending on the type of spondyloarthritis.

### Risk of Bias Assessment

Four longitudinal observational studies were evaluated using the NOS scale (Table 2). One was of medium quality (13), another was of low quality (9) as the other two studies were of high quality. One study showed a possible selection bias as eligibility was not clearly described (9). All studies were matched or adjusted by sex and age but none evaluated possible confounding factors. Only one study also adjusted for race (12). Two studies did not provide information about loss to follow-up (12) or nonresponse rate (9).

**Table 2:** Risk of bias assessment of the cohort and case control studies.

**References**	**Selection**	**Comparability**	**Outcome/Exposure**	**Quality**
Bengtsson et al. (7)	4	1	3	HIGH
Feld et al. (9)	2	1	2	LOW
Ward (12)	4	2	2	HIGH
Yildrir et al. (13)	3	1	3	MEDIUM

None of the four cross sectional studies evaluated possible confounding factors. The statistical method was not clearly described in two studies (8, 11).

### Description and Analysis of the Results

Five studies reported data regarding the risk of spondyloarthritis for bundle branch block, atrioventricular block or pacemaker implantation when compared to the population without spondyloarthritis.

#### Bundle Branch Block

No studies provided information regarding the risk of BBB in patients with SpA. Three studies compared the prevalence of BBB between SpA patients and control groups (6, 11, 13).

Only one or two cases of RBBB and LBBB were observed in the SpA group of each of the studies. The proportion of SpA patients with RBBB was between 0.9% and 1.14%, and the proportion of LBBB was between 1 and 2.7%. The proportion of control groups with RBBB was 0 and 4.1% (*p* = 1.0), and 0 and 2.7% for groups with LBBB (*p* = 0.1). In all studies the comparison of prevalence or the OR were neither clinically nor statistically significant.

#### Atrioventricular Block

Only one study provided information regarding the risk of high grade AVB in SpA (7). Three studies compared the prevalence of low and high grade AVB in SpA and control groups (6, 9, 11, 13).

When using (6, 9, 11) ECG the prevalence of SpA patients with 1st degree AVB was 1–2.8% and no case of 2nd/3rd degree AVB was observed in any of the studies. No case of AVB was observed in the control groups. In all studies the comparison of the prevalence or the OR were neither clinically nor statistically significant (*p* = 1.0).

When using a Holter monitor, transient cases of AVB were observed (13). The prevalence of SpA patients with 1st degree AVB was 3.41%, that of Mobitz II 2nd degree AVB was 1.13% and that of asymptomatic 3rd degree AVB was 1.13%. Only one person from control group had transient Mobitz I 2nd degree AVB but none other type of AVB was observed. Once again, the differences in prevalence and OR were neither clinically nor statistically significant (*p* = 0.56).

The risk of high grade AVB (2nd and 3rd degree) was evaluated using ICD-9 codes by a population based registry (7). The sex and age adjusted IR of AVB was 0.9 (95% CI 0.6–1.3) in AS, 1.2 (95% CI 0.5–1.9) in uSpA and 0.7 (95% CI 0.5–0.9) in PsA. The sex and age adjusted HR of AVB was 2.3 (95% CI 1.6–3.3) in AS, 2.9 (95% CI 1.8–4.7) in uSpA and 1.5 (95% CI 1.1 a 1.9) in PsA.

#### Pacemaker Implantation

Two population based registries evaluated the risk of pacemaker implantation in spondyloarthritis compared to a control group.

The incidence, absolute risk, IR (1,000 person-years) and HR adjusted for age and sex in the Swedish population were reported[|2]. The incidence (1,000 person-years) was 0.86 in AS, 0.61 in uSpA and 0.6 in PsA. The IR was 2.0 (95% CI 1.3–2.7) in AS, 1.5 (95% CI 0.7–2.2) in uSpA and 1.5 (95% CI 1.2–1.8) for PsA. The absolute risk for AVB was 0.4% (moderate to high; 95% CI 0.34–0.69%) for AS, 0.33% (moderate to high; 95% CI 0.21–0.53%) for uSpA and 0.34% (moderate to high; 95% CI 0.26–0.45%) for PsA. The sex and age adjusted HR was 2.0 (95% CI 1.6–2.8) in AS, 1.9 (95% CI 1.2–2.8) in uSpA and 1.6 (95% CI 1.3–1.9) for PsA.

Incidence, absolute risk, OR and RR adjusted for sex and race were reported in an American AS population older than 65 years of age stratified by age intervals (12). The incidence (1,000 person-years) was 3.59 (95% CI 3.26–4.10) for the group aged between 65 and 69 years, 6.68 (95% CI 6.28–7.36) for the 70–75 group, 10.04 (CI 95% 9.66–11.04) for the 75–79 group, 14.6 (95% CI 14.21–15.61) for groups older than 80 years of age. The absolute risk for PMI in AS patients was 0.7% (moderate to high risk; 95% CI 0.6–0.8%) for groups aged between 65 and 69, 1.44% (high risk; 95% CI 1.33–1.6%) for 70–75 years, 2.09% (high risk; 95% CI 2.0–2.2%) for 75–79 years and 4.15% (high risk; 95% CI 4.0–4.3%) for groups older than 80 years. The OR was 1.38 (95% CI 0.97–1.96) for the group aged 65–69 years, 1.28 (95% CI 0.82–1.67) for the 70–75 group, 1.18 (95% CI 0.94–1.48) for the 75–79 group, and 1.23 (95% CI 1.1–1.39) for the group older than 80 years of age. The RR was 1.3 (95% CI 1.16–1.46) for the group aged between 65 and 69 years, 1.33 (95% CI 1.22–1.44) for the 70–75 group, 1.24 (95% CI 1.55–1.33) for the 75–79 group and 1.11 (95% CI 1.06–1.17) for the group older than 80 years of age. The causes for PMI were sinoatrial dysfunction in 41.8%, AVB in 16.6% and atrial fibrillation in 7.6%.

### Spondyloarthritis Related Prognostic Factors Associated to Conduction Disorders

Even though it was not the main objective of this systematic review, we synthesized the data retrieved on SpA disease related prognostic factors for AVB or BBB. Three studies evaluated indirect association (8–10).

Two studies evaluated AVB by studying the possible association between the clinical factors of SpA and PR interval duration (8, 9). PR interval duration was associated with age (*B* = 0.6, *p* = 0.001), disease duration (*B* = 0.75, *p* ≤ 0.001) and BMI (*B* = 1.23, *p* = 0.016) by univariate analysis (8). Age and disease duration was statistically significant by multivariate analysis but no further data was provided. On the contrary another study did not find any correlation with disease duration, subtype of PsA or uveitis but the authors concluded that the sample size was too small to detect possible associations (9).

The prevalence of RBBB was similar in AS patients between 18 and 50 years of age with and without kyphosis due to more evolved disease (10). In patients with kyphosis 3 (5.3%) cases of RBBB were observed while in patients without kyphosis four cases (6.2%) were observed. The differences in prevalences were neither clinically nor statistically significant (*p* = 0.86).

## Discussion

### Summary of the Main Findings

The main findings of this systematic review showed that there are no significant differences in prevalences of BBB and low grade AVB in SpA patients when compared to a control group. The risk of high grade AVB and PMI was approximately double-fold increased in all types of SpA adjusted for sex and age according to a population based registry. The risk of PMI in AS population older than 65 years was smaller lower according to another population based registry possibly because PMI risk is increased in older populations. The RR was about 1.3 but showed a decreasing tendency with older age intervals adjusted for sex and race.

### BBB

Isolated BBB is a conduction delay that does not require treatment in itself (14). The prevalence of BBB is difficult to estimate because it is frequently underdiagnosed in asymptomatic patients. In the Framingham study (15) the prevalence of BBB was between 0.5 and 1% in subjects under 50 years of age but prevalence was almost 11% in subjects between 80 and 90 years of age.

In this systematic review, no study that evaluated risk of BBB in SpA was found. Two cross sectional and case control studies provided indirect evidence that compared prevalence in SpA group and control group. Very few cases were observed in each group. The sample sizes were not adequate for evaluating the low prevalence of BBB in the young population under 65. Also important information that may have shed some light on, such as cardiovascular risk factors, cardiac disease, drugs and SpA disease related characteristics were missing.

### AVB and PM

First degree, Mobitz I and transient AVB are asymptomatic and benign so they may not be detected if not sought. The prevalence of low degree AVB increases at older age (16, 17) and may progress over time to a higher grade AVB. The overall prevalence for low grade and transient AVB was around 1–3%, The prevalence of low grade AVB was similar in control groups according to two case control studies. Once again, due to the scarce number of cases, small sample size and lack of confounder analysis, the results do not allow us to draw conclusions about the risk of SpA for low grade AVB. It would be of interest to evaluate if SpA patients present AVB at a younger age and detect progression to a higher grade AVB through a longitudinal cohort study with a long follow-up.

In contrast, high grade AVB (Mobitz II or 3rd degree) (16) is a potentially life-threatening conduction disease that may lead to a cardiac arrest. High grade AVB usually requires hospitalization for PM implantation (18). Thus a population based registry based on discharge information would adequately reflect the real incidence and risk of high grade AVB if adjusted for appropriate confounders. According to the results of this systematic review, there is a two-fold increase in the risk of high grade AVB in the SpA population compared to the control population according to a population based registry (7). Accordingly, the same study also observed a two-fold increase in the risk of PM in the SpA population compared to the control population (7).

Another registry based on population older than 65 years of age (12) did not observe a relevant risk of PMI (RR between 1.1 and 1.3) compared to the control population. Interestingly, this study also showed a decreasing risk in higher age intervals practically matching the control population in populations older than 80 years of age. High grade AVB and PM implantation are conditions that occur in older age. According to these studies, it may be speculated that in SpA patients high grade AVB and PM implantation occur at younger ages. However when compared to a longitudinal cohort study conducted in a hospital setting, population registry sources lack clinical follow-up information which is essential to study confounders. Results must be adjusted for drugs, cardiovascular risk factors or previous cardiac diseases.

### SpA Related Prognostic Factors for Conduction Disorders

As for possible SpA related prognostic factors for conduction disorders, only one study provided some relevant information. However, this study evaluated indirect outcome measures using the PR interval. The PR interval was associated with age and disease duration when adjusted for in a multiple regression (8). Other studies were not adjusted and had a very small number of cases.

### Strength and Limitations

This systematic review aimed to verify and challenge rooted concepts using an exhaustive bibliographic search of the existing literature without time limit. This is the first systematic review of the literature to evaluate the prevalences and risk of AVB, BBB, and PMI in SpA. Meta-analysis was not carried out due to the heterogeneity of the population, design, diagnostic test and outcome measures of the few studies retrieved. Indirect outcomes were reported such as comparisons of prevalence with the aim of being as comprehensive as possible given the information provided even though most studies lacked sufficient statistical power.

## Conclusions

None of the studies provided results about the risk of low grade AVB and BBB in SpA patients. Indirect results comparing prevalence from low to medium quality studies were found. These studies conclude that there was no difference in prevalence of low grade AVB and BBB in SpA patients when compared to control groups. According to population based registries, a higher risk of high degree AVB and PM implantation in younger aged SpA patients compared to control groups when adjusted by age and sex. However these results were not adjusted by clinically relevant confounders.

The majority of the studies that evaluate the association between cardiac diseases and inflammatory rheumatic diseases are small scaled observational studies or population based registries. Small scaled studies may not have sufficient power to detect differences of an infrequent manifestation but may find small subclinical changes in an asymptomatic stage. Population based registries may be useful for studying infrequent manifestations but lack clinically relevant information to adjust for confounders. Prospective cohort studies with long follow-up are the most accurate design, as for example, the CARMA project (19) for cardiovascular mortality. However this kind of study is rare due to the considerable research effort and budget needed. Among a sea of similar information, it is necessary to critically review the available evidence to date and acknowledge the downsides to guide further investigation of each of the cardiac manifestations and inflammatory rheumatic diseases.

The results obtained in our review give rise to many questions that remain to be answered. We suggest that future research into on conduction disorder in SpA should look deeper into the influence of age and identify which are the prognostic factors. Further studies must analyze the severity of conduction disorders and evaluate the influence of obesity, cardiovascular risk factors and medication in collaboration with cardiologists. From then on studies may evaluate the usefulness of screening (ECG, Holter monitor or even wearable devices) for early detection of conduction disorders in patients that are at higher risk. the, the progression of low grade to higher grade AVB and screening with and identify possible prognostic factors.

## Data Availability Statement

The original contributions presented in the study are included in the article/Supplementary Material, further inquiries can be directed to the corresponding author.

## Author Contributions

MG-R performed the systematic literature search. HP and AL were involved in data screening, extraction, analysis and drafted the manuscript. PD, HC, and MM contributed substantially to the study conceptions, design and critical revision of the article. JS-V and CA-M were consulted for interpretation of the extracted data as referent cardiologists. All authors read and approved the final manuscript.

## Conflict of Interest

AL has received speaker fees/honoraria from Abbvie, Lilly, Novartis, Pfizer and UCB. HC has received speaker fees/honoraria from BMS, Gebro, MSD, Lilly, Novartis, Pfizer, Roche, Sanofi, and UCB, participated in consulting for Abbvie, Amgen, Biogen, Celgene, Gilead, Kern, Pfizer, and Sanofi. The remaining authors declare that the research was conducted in the absence of any commercial or financial relationships that could be construed as a potential conflict of interest.

## Publisher's Note

All claims expressed in this article are solely those of the authors and do not necessarily represent those of their affiliated organizations, or those of the publisher, the editors and the reviewers. Any product that may be evaluated in this article, or claim that may be made by its manufacturer, is not guaranteed or endorsed by the publisher.

## Abbreviations

AS: Ankylosing Spondylitis
AVB, Atrioventricular Block BBB: Bundle Branch Block
BMI: Body Mass Index
ECG, Electrocardiogram ICD, International Classifica IR, Incidence Rate HR, Hazard Ratio LBBB, Left Bundle Branch Block PM, Pacemaker OR: Odds Ratio
ReA: Reactive arthritis
POR, Prevalence Odds Ratio PsA: Psoriatic Arthritis
RBBB, Right Bundle Branch Block RR, Risk Ratio SpA: spondyloarthritis
uSpA: undifferentiated spondyloarthritis.
