# Supplementary material for: Prevalence and Risk for Bundle Branch Block, Atrioventricular Block and Pacemaker Implantation in Spondyloarthritis. A Systematic Review of the Literature
Source: Front Med (Lausanne). 2022 Mar 25;9:851483. doi: 10.3389/fmed.2022.851483 (PMC8990789; doi:10.3389/fmed.2022.851483)
Supplement: Supplementary file 1 [file Table_1.docx]

**Supplementary Table 1. PRISMA statement checklist**

*From:*  Page MJ, McKenzie JE, Bossuyt PM, Boutron I, Hoffmann TC, Mulrow CD, et al. The PRISMA 2020 statement: an updated guideline for reporting systematic reviews. BMJ 2021;372:n71. doi: 10.1136/bmj.n71

| **Section and Topic** | **Item #** | **Checklist item** | **Location where item is reported** |
| --- | --- | --- | --- |
| **TITLE** | | |  |
| Title | 1 | Identify the report as a systematic review. | 1-2 |
| **ABSTRACT** | | |  |
| Abstract | 2 | See the PRISMA 2020 for Abstracts checklist. | 1-2 |
| **INTRODUCTION** | | |  |
| Rationale | 3 | Describe the rationale for the review in the context of existing knowledge. | 2 |
| Objectives | 4 | Provide an explicit statement of the objective(s) or question(s) the review addresses. | 2 |
| **METHODS** | | |  |
| Eligibility criteria | 5 | Specify the inclusion and exclusion criteria for the review and how studies were grouped for the syntheses. | 3 |
| Information sources | 6 | Specify all databases, registers, websites, organisations, reference lists and other sources searched or consulted to identify studies. Specify the date when each source was last searched or consulted. | 3 |
| Search strategy | 7 | Present the full search strategies for all databases, registers and websites, including any filters and limits used. | 3 |
| Selection process | 8 | Specify the methods used to decide whether a study met the inclusion criteria of the review, including how many reviewers screened each record and each report retrieved, whether they worked independently, and if applicable, details of automation tools used in the process. | 3-4 |
| Data collection process | 9 | Specify the methods used to collect data from reports, including how many reviewers collected data from each report, whether they worked independently, any processes for obtaining or confirming data from study investigators, and if applicable, details of automation tools used in the process. | 3-4 |
| Data items | 10a | List and define all outcomes for which data were sought. Specify whether all results that were compatible with each outcome domain in each study were sought (e.g. for all measures, time points, analyses), and if not, the methods used to decide which results to collect. | 4-6 |
|  | 10b | List and define all other variables for which data were sought (e.g. participant and intervention characteristics, funding sources). Describe any assumptions made about any missing or unclear information. | 4-6 |
| Study risk of bias assessment | 11 | Specify the methods used to assess risk of bias in the included studies, including details of the tool(s) used, how many reviewers assessed each study and whether they worked independently, and if applicable, details of automation tools used in the process. | 4 |
| Effect measures | 12 | Specify for each outcome the effect measure(s) (e.g. risk ratio, mean difference) used in the synthesis or presentation of results. | 3-4 |
| Synthesis methods | 13a | Describe the processes used to decide which studies were eligible for each synthesis (e.g. tabulating the study intervention characteristics and comparing against the planned groups for each synthesis (item #5)). | 4-6 |
|  | 13b | Describe any methods required to prepare the data for presentation or synthesis, such as handling of missing summary statistics, or data conversions. | 4 |
|  | 13c | Describe any methods used to tabulate or visually display results of individual studies and syntheses. | 4-6 |
|  | 13d | Describe any methods used to synthesize results and provide a rationale for the choice(s). If meta-analysis was performed, describe the model(s), method(s) to identify the presence and extent of statistical heterogeneity, and software package(s) used. | NA |
|  | 13e | Describe any methods used to explore possible causes of heterogeneity among study results (e.g. subgroup analysis, meta-regression). | NA |
|  | 13f | Describe any sensitivity analyses conducted to assess robustness of the synthesized results. | NA |
| Reporting bias assessment | 14 | Describe any methods used to assess risk of bias due to missing results in a synthesis (arising from reporting biases). | NA |
| Certainty assessment | 15 | Describe any methods used to assess certainty (or confidence) in the body of evidence for an outcome. | NA |
| **RESULTS** | | |  |
| Study selection | 16a | Describe the results of the search and selection process, from the number of records identified in the search to the number of studies included in the review, ideally using a flow diagram. | 4 |
|  | 16b | Cite studies that might appear to meet the inclusion criteria, but which were excluded, and explain why they were excluded. | NA |
| Study characteristics | 17 | Cite each included study and present its characteristics. | 4-6 |
| Risk of bias in studies | 18 | Present assessments of risk of bias for each included study. | 5 |
| Results of individual studies | 19 | For all outcomes, present, for each study: (a) summary statistics for each group (where appropriate) and (b) an effect estimate and its precision (e.g. confidence/credible interval), ideally using structured tables or plots. | NA |
| Results of syntheses | 20a | For each synthesis, briefly summarise the characteristics and risk of bias among contributing studies. | 5 |
|  | 20b | Present results of all statistical syntheses conducted. If meta-analysis was done, present for each the summary estimate and its precision (e.g. confidence/credible interval) and measures of statistical heterogeneity. If comparing groups, describe the direction of the effect. | NA |
|  | 20c | Present results of all investigations of possible causes of heterogeneity among study results. | NA |
|  | 20d | Present results of all sensitivity analyses conducted to assess the robustness of the synthesized results. | NA |
| Reporting biases | 21 | Present assessments of risk of bias due to missing results (arising from reporting biases) for each synthesis assessed. | NA |
| Certainty of evidence | 22 | Present assessments of certainty (or confidence) in the body of evidence for each outcome assessed. | NA |
| **DISCUSSION** | | |  |
| Discussion | 23a | Provide a general interpretation of the results in the context of other evidence. | 6-8 |
|  | 23b | Discuss any limitations of the evidence included in the review. | 8 |
|  | 23c | Discuss any limitations of the review processes used. | 8 |
|  | 23d | Discuss implications of the results for practice, policy, and future research. | 8 |
| **OTHER INFORMATION** | | |  |
| Registration and protocol | 24a | Provide registration information for the review, including register name and registration number, or state that the review was not registered. | NA |
|  | 24b | Indicate where the review protocol can be accessed, or state that a protocol was not prepared. | NA |
|  | 24c | Describe and explain any amendments to information provided at registration or in the protocol. | NA |
| Support | 25 | Describe sources of financial or non-financial support for the review, and the role of the funders or sponsors in the review. | 11 |
| Competing interests | 26 | Declare any competing interests of review authors. | 11 |
| Availability of data, code and other materials | 27 | Report which of the following are publicly available and where they can be found: template data collection forms; data extracted from included studies; data used for all analyses; analytic code; any other materials used in the review. | 11 |

**Supplementary Figure 1. Search strategy**

Search strategy for PubMed:

(("Spondylarthropathies"[Mesh]) OR ("Spondylarthritis"[Mesh]) OR ("Spondylitis, Ankylosing"[Mesh]) OR (“Bechterew Disease”[Title/Abstract]) OR (“Bechterew's Disease”[Title/Abstract]) OR (“Ankylosing Spondyloarthritis”[Title/Abstract]) OR (“Ankylosing Spondylarthritis”[Title/Abstract]) OR (“Ankylosing Spondylitis”[Title/Abstract]) OR (“Spondylarthritis Ankylopoietica”[Title/Abstract]) OR (“Rheumatoid Spondylitis”[Title/Abstract]) OR (“Spondylitis Ankylopoietica”[Title/Abstract]) OR (“Ankylosing Spondyloarthriti*”[Title/Abstract]) OR (Spondyloarthritide*[Title/Abstract]) OR (Spondyloarthriti*[Title/Abstract]) OR (Spondylarthritide*[Title/Abstract]) OR (“Spinal Arthritis”[Title/Abstract]))

AND

(("Arrhythmias, Cardiac"[Mesh]) OR ("Electrocardiography"[Mesh]) OR ("Cardiac Conduction System Disease"[Mesh]) OR ("Heart Conduction System"[Mesh]) OR

("Conduction Disturbances"[Title/Abstract]) OR (“Cardiac Dysrhythmia”[Title/Abstract]) OR (Arrhythm*[Title/Abstract]) OR ("Cardiac Arrhythmias"[Title/Abstract]) OR ("Cardiac Dysrhythmias”[Title/Abstract]) OR ("Sinus Arrhythmia"[Title/Abstract]) OR ("Auricular Fibrillation"[Title/Abstract]) OR ("Atrial Fibrillation"[Title/Abstract]) OR ("Persistent Atrial Fibrillation"[Title/Abstract]) OR ("Auricular Flutter"[Title/Abstract]) OR ("Atrial Flutters"[Title/Abstract]) OR (Bradycard*[Title/Abstract]) OR (Bradyarrhythm*[Title/Abstract]) OR ("Premature Beats"[Title/Abstract]) OR (Extrasystol*[Title/Abstract]) OR ("Auriculo Ventricular Dissociation"[Title/Abstract]) OR

("Heart Blocks"[Title/Abstract]) OR (“Atrioventricular Blocks”[Title/Abstract]) OR (“Fascicular Block”[Title/Abstract]) OR (“Fascicular Blocks”[Title/Abstract]) OR (“Interatrial Block”[Title/Abstract])

OR ("Intraventricular conduction"[Title/Abstract])

OR (“conduction system abnormalit*"[Title/Abstract]) OR (“conduction abnormalit*"[Title/Abstract]) OR (“conduction disorder*"[Title/Abstract]) OR electrocardiogr*[Title/Abstract] OR (“ECG"[Title/Abstract]) OR (“conduction disorder”[Title/Abstract]) OR (bundle*[Title/Abstract]) OR (pacemaker*[Title/Abstract]))

NOT ("Clinical Conference"[Publication Type] OR "Congress"[Publication Type] OR "Consensus Development Conference"[Publication Type] OR "Editorial"[Publication Type] OR "Published Erratum"[Publication Type] OR "Letter"[Publication Type] OR "Comment"[Publication Type])

NOT ("Animals"[Mesh] NOT ("Animals"[Mesh] AND "Humans"[Mesh]))

Filters: From 2021

Search strategy for EMBASE

('spondyloarthropathy'/exp OR 'spondylarthritis'/exp OR 'ankylosing spondylitis'/exp OR 'bechterew disease*':ab,ti OR 'ankylosing spondyloarthriti*':ab,ti OR 'ankylosing spondylarthriti*':ab,ti OR 'ankylosing spondylitis':ab,ti OR 'spondylarthritis ankylopoietica':ab,ti OR 'Rheumatoid Spondyliti*':ab,ti OR 'Spondylitis Ankylopoietica':ab,ti OR 'Ankylosing Spondyloarthriti*':ab,ti OR Spondyloarthritid*:ab,ti OR Spondyloarthritis*:ab,ti OR Spondylarthritid*:ab,ti OR 'spinal arthritis':ab,ti OR 'spondylosis'/exp)

AND

('heart arrhythmia'/exp OR "Electrocardiography"/exp OR 'heart muscle conduction disturbance'/exp OR 'heart muscle conduction system'/exp OR 'Conduction Disturbanc*':ab,ti OR 'Cardiac Dysrhythmia*':ab,ti OR Arrhythm*:ab,ti OR 'Cardiac Arrhythmia*':ab,ti OR 'Cardiac Dysrhythmias':ab,ti OR 'Sinus Arrhythmia':ab,ti OR 'Auricular Fibrillation*':ab,ti OR 'Paroxysmal Atrial Fibrillation':ab,ti OR 'Persistent Atrial Fibrillation*':ab,ti OR 'Auricular Flutter*':ab,ti OR 'Atrial Flutters':ab,ti OR Bradycard*:ab,ti OR Bradyarrhythm*:ab,ti OR 'Premature Beats':ab,ti OR Extrasystol*:ab,ti OR 'Auriculo Ventricular Dissociation*':ab,ti OR 'Heart Block*':ab,ti OR 'Atrioventricular Blocks':ab,ti OR 'AV Block':ab,ti OR 'Bundle Branch Block':ab,ti OR 'Fascicular Block':ab,ti OR 'Right Bundle Branch Block':ab,ti OR 'Left Bundle Branch Block':ab,ti OR 'Posterior Fascicular Block':ab,ti OR 'Anterior Fascicular Block':ab,ti OR 'Interatrial Block':ab,ti OR 'Intraventricular conduction':ab,ti OR 'Intraventricular conduction disturbance':ab,ti OR 'Intraventricular conduction time':ab,ti)

AND ([embase]/lim NOT [medline]/lim) NOT ('animals'/exp NOT ('animals'/exp AND 'humans'/exp)) NOT ([conference abstract]/lim OR [conference paper]/lim OR [conference review]/lim OR [erratum]/lim OR [letter]/lim OR [note]/lim OR [short survey]/lim) AND [2021-2021]/py

Search strategy for Cochrane Library:

ID Search Hits

#1 MeSH descriptor: [Spondylarthropathies] explode all trees 1265

#2 MeSH descriptor: [Spondylarthritis] explode all trees 1429

#3 MeSH descriptor: [Spondylitis, Ankylosing] explode all trees 725

#4 ("Bechterew Disease" or "Bechterew's Disease" or "Ankylosing Spondyloarthritis" or "Ankylosing Spondylarthritis" or "Ankylosing Spondylitis" or "Spondylarthritis Ankylopoietica" or Spondylitis or "Spondylitis Ankylopoietica" or "Ankylosing Spondyloarthritis" or Spondyloarthritid* or Spondyloarthrit* or Spondylarthritid* or "Spinal Arthritis"):ab,ti,kw 2824

#5 #1 OR #2 OR #3 OR #4 3349

#6 MeSH descriptor: [Arrhythmias, Cardiac] explode all trees 10094

#7 MeSH descriptor: [Electrocardiography] explode all trees 8957

#8 MeSH descriptor: [Cardiac Conduction System Disease] explode all trees 3038

#9 MeSH descriptor: [Heart Conduction System] explode all trees 915

#10 ("Conduction Disturbances" or "Cardiac Dysrhythmia" or Arrhythm* or "Cardiac Arrhythmias" or "Cardiac Dysrhythmias" or "Sinus Arrhythmia" or "Auricular Fibrillation" or "Atrial Fibrillation" or "Persistent Atrial Fibrillation" or "Auricular Flutter" or "Atrial Flutters" or Bradycard* or Bradyarrhythm* or "Premature Beats" or Extrasystol* or "Auriculo Ventricular Dissociation" or "Heart Blocks" or "Atrioventricular Blocks" or "Fascicular Block" or "Fascicular Blocks" or "Interatrial Block" or "Intraventricular conduction" or "conduction system abnormalities" or "conduction abnormalities" or "conduction disorders" or electrocardiogr* or "ECG" or "conduction disorder" or bundle* or pacemaker*):ab,ti,kw 56933

#11 #6 OR #7 OR #8 OR #9 OR #10 57933

#12

**Supplementary Figure 2. Articles not available in library sources.**

1. Bouvrain Y, Person P, Sikorav H. [Atrioventricular blocks and ankylosing spondylitis]. Annales de cardiologie et d'angeiologie. 1974;23(2):131-5.

2. Follath F, Steiger U, Burkart F. Disorders of auriculoventricular and intraventricular conduction in the course of rheumatoid affections. Medecine et Hygiene. 1973;31(1083):1974-6.

3. Gouffault J, Boudesseul B, Courgeon P, Bourel M. [Intracardiac conduction disorders in ankylosing spondylarthritis]. Archives des maladies du coeur et des vaisseaux. 1972;65(2):226-34.

4. Julkunen H. Atrioventricular conduction defect in ankylosing spondylitis. Geriatrics. 1966;21(10):129-31.

**Supplementary Figure 3. Excluded articles after full text reading.**

| **Reference** | **Exclusion criteria** |
| --- | --- |
| Alexander, B et al.[1] | Wrong design |
| Atzeni, F et al. [2] | Wrong design |
| Badui-Dergal, E et al [3] | Insufficient sample size |
| Ben Taarit, C et al. [4] | Wrong outcome |
| Bergfeldt, L et al. [5] | Wrong design |
| Bergfeldt, L et al. [6] | Wrong population |
| Bottiger, LE et al. [7] | Wrong design |
| Bremander, A et al [8] | Wrong outcome |
| Breunig, M et al [9] | Wrong outcome |
| Brunner, F et al [10] | Wrong outcome |
| Castañeda, S et al [11] | Wrong design |
| Castañeda, S et al [12] | Wrong design |
| Chan, S et al [13] | Wrong outcome |
| Chiu, HY et al [14] | Wrong outcome |
| Cliff, JM et al [15] | Wrong outcome |
| Csonka, GW et al [16] | Wrong design |
| Csonka, GW et al [17] | Wrong design |
| Deer, T et al [18] | Wrong design |
| Descalzo, M et al [19] | Wrong outcome |
| Eddarami, J et al [20] | Insufficient data |
| Forsblad-d’Elia, H et al [21] | Wrong outcome |
| Gensler, LS [22] | Wrong design |
| Gijon Baños, J et al [23] | Insufficient data |
| Ho, H et al [24] | Wrong design |
| Julkunen, H et al [25] | Wrong design |
| Klingberg, E et al [26] | Wrong design |
| Lautermann, D et al [27] | Wrong design |
| Ljung, L et al [28] | Wrong outcome |
| Moller, P [29] | Wrong outcome |
| Morovatdar, N et al [30] | Wrong outcome |
| Nagyhegyi, G et al [31] | Wrong design |
| Palazzi, G et al [32] | Wrong outcome |
| Vazquez-García, R et al [33] | Insufficient data |

1. Alexander B, Feiner H. Ankylosing spondylitis with cardiac dysrhythmia; pathologic changes in cardiac conduction system. New York state journal of medicine. 1979;79(10):1585-8.

2. Atzeni F, Corda M, Sarzi-Puttini P, Caso F, Turiel M. From old to new cardiovascular complications in ankylosing spondylitis. Israel Medical Association Journal. 2017;19(8):506-9.

3. Badui-Dergal E. [The heart in various connective tissue diseases]. Gaceta medica de Mexico. 1990;126(3):175-87; discussion 88-9.

4. Ben Taarit C, Kaffel D, Ben Maiz H, Khedher A. [Cardiovascular manifestations in ankylosing spondylitis. Concerning 210 cases]. La Tunisie medicale. 2008;86(6):546-9.

5. Bergfeldt L. HLA-B27-associated cardiac disease. Annals of internal medicine. 1997;127(8 Pt 1):621-9.

6. Bergfeldt L, Allebeck P, Edhag O. Mortality in pacemaker-treated patients. A follow-up study of the impact of HLA B27 and associated rheumatic disorders. Acta medica Scandinavica. 1987;222(4):293-9.

7. Bottiger LE, Edhag O. Heart block in ankylosing spondylitis and uropolyarthritis. British heart journal. 1972;34(5):487-92.

8. Bremander A, Petersson IF, Bergman S, Englund M. Population-based estimates of common comorbidities and cardiovascular disease in ankylosing spondylitis. Arthritis care & research. 2011;63(4):550-6.

9. Breunig M, Kleinert S, Lehmann S, Kneitz C, Feuchtenberger M, Tony HP, et al. Simple screening tools predict death and cardiovascular events in patients with rheumatic disease. Scandinavian journal of rheumatology. 2017:1-8.

10. Brunner F, Kunz A, Weber U, Kissling R. Ankylosing spondylitis and heart abnormalities: do cardiac conduction disorders, valve regurgitation and diastolic dysfunction occur more often in male patients with diagnosed ankylosing spondylitis for over 15 years than in the normal population? Clinical rheumatology. 2006;25(1):24-9.

11. Castaneda S, Gonzalez-Juanatey C, Gonzalez-Gay MA. Inflammatory Arthritis and Heart Disease. Current pharmaceutical design. 2018.

12. Castañeda S, González-Juanatey C, González-Gay MA. Sex and Cardiovascular Involvement in Inflammatory Joint Diseases. Clinical Reviews in Allergy and Immunology. 2017:1-15.

13. Chan SCW, Teo CK, Li PH, Lau KK, Lau CS, Chung HY. Cardiovascular risk in patients with spondyloarthritis and association with anti-TNF drugs. Therapeutic Advances in Musculoskeletal Disease. 2021;13.

14. Chiu HY, Chang WL, Huang WF, Wen YW, Tsai YW, Tsai TF. Increased risk of arrhythmia in patients with psoriatic disease: A nationwide population-based matched cohort study. Journal of the American Academy of Dermatology. 2015;73(3):429-38.

15. Cliff JM. Spinal bony bridging and carditis in Reiter's disease. Annals of the rheumatic diseases. 1971;30(2):171-9.

16. Csonka GW. Workshop I. Features and prognosis of Reiter's syndrome. Clinical aspects of Reiter's syndrome. Annals of the rheumatic diseases. 1979;38 Suppl 1:suppl 4-7.

17. Csonka GW, Oates JK. Pericarditis and electrocardiographic changes in Reiter's syndrome. British medical journal. 1957;1(5023):866-9.

18. Deer T, Rosencrance JG, Chillag SA. Cardiac conduction manifestations of Reiter's syndrome. Southern medical journal. 1991;84(6):799-800.

19. Descalzo MÁ, Montero D, Erra A, Marsal S, Fernández Castro M, Mulero J, et al. Spanish registry for adverse events of biological therapy in rheumatic diseases (BIOBADASER): State report, January 26th, 2006. Reumatologia Clinica. 2007;3(1):4-20.

20. Eddarami J, Azzouzi H, Ichchou L. Heart Involvement in a Moroccan Population with Spondyloarthritis: A Cross-sectional Study. Journal of the Saudi Heart Association. 2021;33(2):191-7.

21. Forsblad-d'Elia H, Wallberg H, Klingberg E, Carlsten H, Bergfeldt L. Cardiac conduction system abnormalities in ankylosing spondylitis: a cross-sectional study. BMC musculoskeletal disorders. 2013;14:237.

22. Gensler LS. Axial spondyloarthritis: the heart of the matter. Clinical rheumatology. 2015;34(6):995-8.

23. Gijon Banos J, Garcia Fernandez F, Vesga Carasa JC, Balsa Criado A, de Miguel Mendieta E, Pavon C, et al. [Ankylosing spondylitis and cardiopathy]. Revista clinica espanola. 1987;181(6):323-6.

24. Ho HH, Chen JY. Ankylosing spondylitis: Chinese perspective, clinical phenotypes, and associated extra-articular systemic features. Current rheumatology reports. 2013;15(8):344.

25. Julkunen H, Luomanmaeki K. COMPLETE HEART BLOCK IN RHEUMATOID (ANKYLOSING) SPONDYLITIS. Acta medica Scandinavica. 1964;176:401-5.

26. Klingberg E, Svealv BG, Tang MS, Bech-Hanssen O, Forsblad-d'Elia H, Bergfeldt L. Aortic Regurgitation Is Common in Ankylosing Spondylitis: Time for Routine Echocardiography Evaluation? The American journal of medicine. 2015;128(11):1244-50.e1.

27. Lautermann D, Braun J. Ankylosing spondylitis--cardiac manifestations. Clinical and experimental rheumatology. 2002;20(6 Suppl 28):S11-5.

28. Ljung L, Sundstrom B, Smeds J, Ketonen M, Forsblad-d'Elia H. Patterns of comorbidity and disease characteristics among patients with ankylosing spondylitis-a cross-sectional study. Clinical rheumatology. 2017.

29. Moller P. Atrioventricular conduction time in ankylosing spondylitis. Distribution of P-R intervals in patients and their relatives. Acta medica Scandinavica. 1985;217(1):85-8.

30. Morovatdar N, Watts GF, Bondarsahebi Y, Goldani F, Rahmanipour E, Rezaee R, et al. Ankylosing Spondylitis and Risk of Cardiac Arrhythmia and Conduction Disorders: A Systematic Review and Meta-analysis. Current cardiology reviews. 2021;17(5):e150521193326.

31. Nagyhegyi G, Nadas I, Banyai F, Luzsa G, Geher P, Molnar J, et al. Cardiac and cardiopulmonary disorders in patients with ankylosing spondylitis and rheumatoid arthritis. Clinical and experimental rheumatology. 1988;6(1):17-26.

32. Palazzi C, Salvarani C, D'Angelo S, Olivieri I. Aortitis and periaortitis in ankylosing spondylitis. Joint, bone, spine : revue du rhumatisme. 2011;78(5):451-5.

33. Vazquez Garcia R, Martinez Martinez A, Martinez de la Concha L, Pastor Torres L, Gutierrez de la Pena J. [Cardiovascular manifestations in ankylosing spondylitis]. Revista espanola de cardiologia. 1983;36(3):205-12.
